# Fucosylated Proteome Profiling Identifies a Fucosylated, Non-Ribosomal, Stress-Responsive Species of Ribosomal Protein S3

**DOI:** 10.3390/cells10061310

**Published:** 2021-05-25

**Authors:** Gregory Watson, Daniel Lester, Hui Ren, Connor M. Forsyth, Elliot Medina, David Gonzalez Perez, Lancia Darville, Jiqiang Yao, Vince Luca, John Koomen, Ling Cen, Eric Lau

**Affiliations:** 1 Department of Tumor Biology, Moffitt Cancer Center, Tampa, FL 33602, USA; Gregory.Watson@moffitt.org (G.W.); daniel.lester@moffitt.org (D.L.); huiren.email@gmail.com (H.R.); forsyth.connor@gmail.com (C.M.F.); 2 Department of Drug Discovery, Moffitt Cancer Center, Tampa, FL 33602, USA; Elliot.Medina@moffitt.org (E.M.); David.GonzalezPerez@moffitt.org (D.G.P.); Vince.Luca@moffitt.org (V.L.); 3 Proteomics and Metabolomics Core, Moffitt Cancer Center, Tampa, FL 33602, USA; Lancia.Darville@moffitt.org (L.D.); John.Koomen@moffitt.org (J.K.); 4 Biostatistics and Bioinformatics Shared Resource, Moffitt Cancer Center, Tampa, FL 33602, USA; Jiqiang.Yao@moffitt.org (J.Y.); Ling.Cen@moffitt.org (L.C.); 5 Department of Molecular Oncology, Moffitt Cancer Center, Tampa, FL 33602, USA

**Keywords:** ribosomal protein S3, fucosylation, melanoma

## Abstract

**Simple Summary:**

Dysregulated fucosylation has been characterized as an underlying cause or a contributor to the pathogenesis of several disease states. However, to date, there is not a clear understanding of how and what proteins, signaling pathways, and cellular processes are impacted by fucosylation. Here, we characterized the proteins recognized by a fucose-binding lectin and unexpectedly discovered that many intracellular proteins are putatively subject to posttranslational fucosylation. We further found that fucosylation on intracellular ribosomal protein S3 responds to stimulus, and that it appears to be independent of the currently characterized fucosylation pathway. This work suggests a to-date-underappreciated role for fucosylation on intracellular proteins and supports the existence of fucosylation capabilities within cells that is not fully known.

**Abstract:**

Alterations in genes encoding for proteins that control fucosylation are known to play causative roles in several developmental disorders, such as Dowling-Degos disease 2 and congenital disorder of glycosylation type IIc (CDGIIc). Recent studies have provided evidence that changes in fucosylation can contribute to the development and progression of several different types of cancers. It is therefore important to gain a detailed understanding of how fucosylation is altered in disease states so that interventions may be developed for therapeutic purposes. In this report, we find that fucosylation occurs on many intracellular proteins. This is an interesting finding, as the fucosylation machinery is restricted to the secretory pathway and is thought to predominately affect cell-membrane-bound and secreted proteins. We find that Ribosomal protein S3 (RPS3) is fucosylated in normal tissues and in cancer cells, and that the extent of its fucosylation appears to respond to stress, including MAPK inhibitors, suggesting a new role in posttranslational protein function. Our data identify a new ribosome-independent species of fucosylated RPS3 that interacts with proteins involved in posttranscriptional regulation of RNA, such as Heterogeneous nuclear ribonucleoprotein U (HNRNPU), as well as with a predominance of non-coding RNAs. These data highlight a novel role for RPS3, which, given previously reported oncogenic roles for RPS3, might represent functions that are perturbed in pathologies such as cancer. Together, our findings suggest a previously unrecognized role for fucosylation in directly influencing intracellular protein functions.

## 1. Introduction

Glycosylation, the posttranslational modification of proteins and lipids with sugars, plays an important role in regulating protein–protein interactions, protein–ligand signaling, and cellular behaviors, particularly during development [1,2,3]. The biological importance of glycosylation can be most appreciated in the number of developmental disorders that result from defects in genes encoding for glycosyltransferases or other proteins with integral roles in the various glycosylation pathways (e.g., nucleotide sugar transporters). Growing evidence has highlighted active roles that alterations in glycosylation in post-developmental disease states (e.g., cancer) can play in disease development and/or progression. In either context, interventions designed to modify or target defects or alterations in glycosylation in disease states have shown promise as therapeutic strategies.

Fucosylation, the posttranslational modification of proteins or lipids with the sugar L-fucose, has recently been shown to be altered during the progression of melanoma and pancreatic cancer [4,5,6]. In melanoma, global fucosylation is altered during disease progression, suggesting that alterations in the fucosylation pathway play a role in disease progression. Indeed, we and others have reported that fucosylation can regulate melanoma motility, although the precise underlying molecular mechanisms remain unclear. Characterizing the substrates that are subject to fucosylation and understanding how fucosylation influences their function, downstream signaling, and ultimately cellular behavior, are expected to highlight how and in which context(s) fucosylation might be exploited as a viable therapeutic target or to identify the key proteins and cellular signaling pathways that respond to alterations in fucosylation and may themselves be targeted for therapeutic intervention.

Here, we characterized melanoma proteins that are recognized by UEAI lectin, a sugar-binding protein that recognizes fucose-containing glycans. We found that UEAI lectin recognizes almost exclusively intracellular proteins, an unexpected observation suggesting that fucosylation can occur on intracellular proteins independent of the canonical fucosylation pathway. We identified and validated ribosomal protein S3 (RPS3) as a fucosylated protein in human cancer cells as well as in normal mouse tissues. We determined that the fucosylated species is ribosome-independent, instead interacting with a group of cytosolic proteins that posttranscriptionally regulate RNAs. This report suggests the existence of an as yet uncharacterized fucosylation pathway that is altered in disease states and that plays a role in regulating intracellular protein function.

## 2. Materials and Methods

### 2.1. Cell Lines and Reagents

All cell lines used in this manuscript were validated for identity by the H. Lee Moffitt Cancer Center Molecular Genomics Core within 2 years of testing and were confirmed to be mycoplasma-free using the Plasmo Test kit (InvivoGen, San Diego, CA, USA). Cell lines were cultured in DMEM (VWR, Radnor, PA, USA) supplemented with heat inactivated fetal bovine serum 1:10 (Peak Serum, Denver, CO, USA). Antibodies: RPS3 (Bethyl, Montgomery, TX, USA), HNRNPU (ThermoFisher, Waltham, MA, USA), BCLAF1 (Santa Cruz Biotechnology, Inc., Santa Cruz, CA, USA), EIF3K (Bethyl), FLAG (Sigma, St. Louis, MO, USA), β-actin (Sigma), β-tubulin (Developmental Studies Hybridoma Bank, DSHB, Iowa City, IA, USA), LaminA/C (DSHB). UEAI, AAL, UEAI-488, AAL-488, UEAI-biotin, AAL-biotin were purchased from Vector Laboratories (Burlingame, CA, USA). Secondary antibodies: anti-rabbit-HRP (Santa Cruz Biotechnology, Inc.), m-IgGκ-BP-HRP (Santa Cruz Biotechnology, Inc.), streptavidin-800 (Life Technologies, Carlsbad, CA, USA), IRDye 680RD anti-mouse (LI-COR, Lincoln, NE, USA).

### 2.2. Plasmids and Cloning

RPS3 coding sequence was cloned into pLENTI-C-MYC-DDK-IRES-puro plasmid (Origene, Rockville, MD, USA), transformed into Stbl3 bacterial cells, and selected on chloramphenicol plates (50 µg/mL) following standard protocols. Colonies were picked, grown, and miniprepped (Qiagen, Hilden, Germany) for sequence verification by Eton Bioscience. Lentiviral particles were produced in 293T cells with VSVG and Δ8.9 plasmids following standard protocols. Cells were transduced and selected on puromycin (1 µg/mL) for stable line expression. pcDNA3.1 MCS-BirA (R118G)-HA plasmid for RPS3 BioID was purchased from Addgene (Watertown, MA, USA) (#36047). MYC-DDK (mDDK; DDK is also known as FLAG) tagged RPS3 was cloned into pCDNA3.1 plasmid for mutagenesis. Mutagenesis was carried out using the Q5 Site-Directed Mutagenesis Kit following the manufacturer’s instructions (NEB, Ipswich, MA, USA).

Primers for constructing RPS3 Δ129-160:

5-GGAGACCCTGTTAACTAC-3

5-CTCCATGATGAACCGCAG-3

### 2.3. Western Blot

Western blotting was carried out following standard protocols. Briefly, cells were washed in cold PBS and harvested for lysate in IP Lysis Buffer (50 mM Tris-HCl pH 8.0, 150 mM NaCl, 2 mM EDTA, 1% Triton X-100) or RIPA Lysis Buffer (50 mM Tris-HCl pH 8.0, 150 mM NaCl, 2 mM EDTA, 1% Triton X-100, 0.5% sodium deoxycholate, 0.1% SDS) supplemented with protease and phosphatase inhibitor tablets (ThermoFisher, Waltham, MA, USA) where appropriate. For cell fractions, cells were harvested in 0.5% Triton X-100 PBS supplemented with protease and phosphatase inhibitor on ice for 10 min (m). Lysates were then spun down at 12,000× *g* for 5 m at 4 °C. Supernatant was removed (cytoplasmic fraction) and Triton-X insoluble material was then lysed/solubilized in RIPA buffer (nuclear fraction). Lysates were then subjected to sonication using a probe sonicator or DNA was sheared using a Dounce homogenizer. Mouse tissue was ground in RIPA Lysis Buffer using a mortar and pestle and further processed using a Dounce homogenizer. Lysates were then cleared of insoluble material by spinning at 12,000× *g* for 5 m at 4 °C. Cell fractionation was carried out by incubating cells on ice for 20 m in 0.5% Triton X-100 PBS supplemented with protease and phosphatase inhibitor tablets (ThermoFisher). Cells were then vortexed for 10 s, and Triton-insoluble material was pelleted by centrifugation at 12,000× *g* for 5 m at 4 °C. Supernatant was then removed and RIPA Lysis Buffered applied to the pellet. Protein concentration was determined using DC Protein Assay (Bio-Rad Laboratories, Hercules, CA, USA). Equal amounts of protein then prepared in 4× Laemmli Buffer (62.5 mM Tris-HCl pH 6.8, 10% glycerol, 0.005% Bromophenol blue, 10% beta-mercaptoethanol) and heated for 10 m at 95 °C prior to SDS-PAGE gel loading. Proteins were blotted onto PVDF membrane (Bio-Rad Laboratories) and blocked in 5% BSA PBST, 5% non-fat dried milk PBST, or Carbo-Free blocking solution (Vector Laboratories) where appropriate. Membranes were then probed for protein of interest overnight at 4 °C. Membranes were then washed in PBST and secondary reagent applied for 1–2 h at room temperature (RT). Membranes were then developed by film or imaged on a LI-COR Odyssey Fc Imaging System where appropriate.

### 2.4. PCR and qRT-PCR

Cells were washed in cold PBS and then RNA was harvested using the GeneElute Mammalian Total RNA Kit (Sigma) or using TRIzol (ThermoFisher) following the manufacturer’s instructions. RNA was quantitated on a NanoDrop and reverse transcribed to cDNA using High-Capacity cDNA Reverse Transcription Kit (ThermoFisher). cDNA was analyzed by PCR using Phusion High-Fidelity DNA Polymerase (NEB) or by qRT-PCR using FastStart SYBR Green Master Mix (Roche, Basel, Switzerland) and quantitated on a CFX96 Touch Real-Time PCR Detection System (Bio-Rad Laboratories).

qRT-PCR primers:

18S rRNA

5-GTAACCCGTTGAACCCCATT-3

5-CCATCCAATCGGTAGTAGCG-3

HES1

5-AAACCAAAGACAGCATCTGAGC-3

5-TTCCCCAGCACACTTGGGTC-3

HOXC9

5-GGGAGGGTTCAGTGTTGAGA-3

5-GGGATGACCTGGACCAAATA-3

HOXC9 Intron

5-TGGGCATCTCCCCAGATTAGA-3

5-GTATAATTAGGCCCTGGCCCC-3

PARP1

5-GGTACCATCAGGCTGCTTT-3

5-TTCGCCACTTCATCCACTCC-3

PTEN

5-AGCTGGAAAGGGACGAACTG-3

5-CCTTTAGCTGGCAGACCACA-3

Histone H3

5-AAGCAGACTGCCCGCAAAT-3

5-GGCCTGTAACGATGAGGTTTC-3

### 2.5. Immunoprecipitation for Protein, RNA, and Mass Spectrometry Analysis

Cells were washed in PBS and then lysed in ice cold IP Lysis Buffer or RIPA Lysis Buffer using a Dounce homogenizer. Lysates were then cleared of insoluble material by spinning at 12,000× *g* for 5 m at 4 °C. Protein concentration was determined using DC Protein Assay (Bio-Rad Laboratories). Lysate concentrations were equalized using IP Lysis Buffer. Equal amounts of protein were incubated overnight at 4 °C with end-over-end rotation with anti-FLAG-agarose (Sigma), lectin-agarose conjugates (Vector Labs), or indicated control (normal mouse IgG or agarose bead). The next day, immunoprecipitations were washed in ice cold 0.1% Triton X-100 PBS. For protein, the immunoprecipitations were reconstituted in Western sample buffer (for Western blotting). For RNA, the samples were reconstituted in TRIzol following the manufacturer’s instructions (for PCR, qRT-PCR, or for analysis by Chip (RIP-Chip). For RIP-Chip, unbiased identification of RNAs bound by RPS3 was carried out by the Molecular Genetics Core Facility at Moffitt Cancer Center on a Clariom D Array (Applied Biosystems, Foster City, CA, USA) following the manufacturer’s instructions. Data were analyzed on the Transcriptome Analysis Console (TAC) following the manufacturer’s instructions. Cutoff for RNAs bound by RPS3 was set at ≤ 2-fold enriched in RPS3 IP relative to control IP. For mass spectrometry, samples were reconstituted in PBS and submitted to the Proteomics Core Facility at Moffitt Cancer Center for processing and analysis. For IP elution and subsequent lectin PD, washed FLAG IP protein complexes were eluted with 3× FLAG Peptide following the manufacturer’s instructions (Sigma). FLAG agarose was then removed by centrifugation, and eluted complexes were then incubated with lectin-agarose conjugates or control beads as described above.

### 2.6. Analysis by LC-MS/MS Mass Spectrometry

Gel bands between ~10 and 50 kDa and from ~50 to 250 were excised and treated with Tris (2-carboxy-ethyl) phosphine hydrochloride (TCEP) and iodoacetamide. In-gel digestion using trypsin was performed overnight at 37 °C. Tryptic peptides were analyzed using nanoflow liquid chromatography (U3000, Dionex, Sunnyvale, CA, USA) coupled to an electrospray bench top orbitrap mass spectrometer (Q-Exactive plus, ThermoFisher) in a data-dependent manner for tandem mass spectrometry peptide sequencing. The peptide mixtures were separated on a 75 µm ID × 25 cm, 2 µm, 100 Å, C18 analytical column (New Objective, Woburn, MA, USA) using a 90 m program at a flow rate of 300 nL/m of 5% solvent B for 8 m, 5% to 38.5% solvent B over 60 m, then 50% to 90% solvent B over 7 m and held at 90% for 5 m, followed by 90% to 5% solvent B in 1 m and re-equilibrated for 10 m. Solvent A was composed of 98% ddH2O and 2% acetonitrile containing 0.1% FA. Solvent B was 90% acetonitrile and 10% ddH2O containing 0.1% FA. Sixteen tandem mass spectra were collected in a data-dependent manner following each survey scan. Data analysis was performed using MASCOT and SEQUEST against the Human UniProt database (downloaded April 2016) to identify proteins from IP samples. The raw files were processed using select parameters, including at least 7 amino acids per peptide, as many as 3 missed cleavages, and a false discovery rate of 0.01 was selected for peptides and proteins. Methionine oxidation and peptide N-terminal acetylation were selected as variable modifications. Both MASCOT [7] and SEQUEST [8] search results were summarized in Scaffold 4.7.3 (Proteome Software, http://www.proteomesoftware.com; accessed date: 30 January 2017).

### 2.7. Immunofluorescence

Cells were cultured and treated on glass coverslips in a 6-well tissue culture dish. Cells were washed in cold PBS and fixed in 4% formaldehyde PBS at RT for 10 m or in ice cold methanol at −20 °C for 15 m. Fixed cells were washed in PBS. Fixed cells were blocked/permeabilized in 5% BSA 0.3% Triton X-100 PBS for 1 h at RT. Primary antibody (as indicated) diluted in blocking buffer was applied ON at 4 °C. The next day, cells were washed in PBS, and fluorophore-conjugated secondary antibody (Invitrogen, Carlsbad, CA, USA) diluted in blocking buffer was applied for 1 h at RT. Cells were washed in PBS and then mounted on glass sides using Prolong Gold Antifade Mountant (ThermoFisher). Cells were analyzed and imaged on a Keyence BZ-700 fluorescent microscope.

### 2.8. Tissue Microarray Staining

Melanoma tissue microarray (TMA) was purchased from US Biomax (Derwood, MD, USA) (Cat. No. ME1004f). Paraffin was melted away by heating at ~70 °C for 30 m before rehydrating the tissue through xylene and ethanol dilution rinses. Antigen retrieval was conducted using DAKO Target Retrieval Solution (Agilent, Santa Clara, CA, USA) following the manufacturer’s instructions. Tissue was blocked using DAKO Protein Block, Serum Free reagent (Agilent). The TMA was probed for RPS3 and Phalloidin-FITC (Invitrogen) overnight at 4 °C. The next day, the TMA was washed and incubated with secondary antibody for 2 h at RT. The slide was then washed 1 time in PBS containing DAPI (ThermoFisher), followed by another 3 washes in PBS. A coverslip was then mounted over the tissue using ProLong Gold Antifade Reagent (ThermoFisher). The tissue slide was imaged at the Moffitt Cancer Center Clinical Testing Department Core Facility, and the signal was quantitated in an unbiased manner using the HALO platform.

### 2.9. Flow Cytometry

Subconfluent cells were harvested in trypsin (Corning Inc., Corning, NY, USA) and washed in cold PBS. Cells were fixed in 4% formaldehyde PBS for 10 m at RT and then washed in PBS. Non-permeabilized cells were then blocked in 5% BSA PBS and incubated with 0.2 µg/mL fluorophore-conjugated lectin (Vector Laboratories) for 1 h at 4 °C. For permeabilization and staining, cells were incubated in 5% BSA 0.3% Triton X-100 PBS for 30–60 m at RT and then incubated with 0.2 µg/mL fluorophore-conjugated lectin (Vector Laboratories) for another 1 h at RT. Stained cells were then washed in PBS twice before being subjected to flow cytometry. Samples were analyzed using FlowJo software (BD Biosciences, East Rutherford, NJ, USA), and statistical analyses were performed on Prism (GraphPad, San Diego, CA, USA).

### 2.10. Biotinylation Identification (BioID)

RPS3 coding sequence was cloned into pCDNA3.1-MCS-BirA (R118G)-HA plasmid (Addgene #36047). The 293T cells were transfected for RPS3-BioID using Lipofectamine 2000 reagent following the manufacturer’s protocol (ThermoFisher), and cells were then cultured for 2 days in media supplemented with biotin prior to lysis. Cells were washed in cold PBS and lysed in IP Lysis Buffer using a Dounce homogenizer. Lysates were cleared on insoluble material by centrifugation at 12,000× *g* for 5 m at 4 °C. Cell lysates were then incubated with neutravidin-conjugated agarose (ThermoFisher) or control agarose beads ON at 4 °C with end-over-end rotation. The next day, IPs were washed in 0.1% Triton X-100 PBS, and the pelleted agarose-IP conjugates were submitted to the Proteomics Core Facility at Moffitt Cancer Center for processing and analysis. Proteins identified with 2 or more exclusive unique spectrum counts were considered as a positive ID. Proteins identified were analyzed and visualized by String (http://string-db.org; accessed date: 4 April 2019) using default parameters.

### 2.11. Click-Chemistry Identification of RPS3 Fucosylation

Click-IT Protein Reaction Buffer Kit and Click-IT Fucose Alkyne were purchased from ThermoFisher and used in accordance with the manufacturer’s instructions. Briefly, 1205LU cells were cultured in media supplemented with or without 50 µM Fucose Alkyne for two days. Cells were subsequently washed in cold PBS and lysed in IP Lysis Buffer using a Dounce homogenizer. Lysates were cleared of insoluble material by centrifugation at 12,000× *g* for 5 m at 4 °C. Lysates were then incubated with biotin azide and the click chemistry reaction was performed following the kit instructions. Proteins were then precipitated in methanol/chloroform. Precipitated proteins were washed thoroughly in methanol and resuspended in 1% SDS with heat. Solubilized protein samples were then diluted in IP Lysis Buffer, followed by removal of a small aliquot to hold in reserve as “Input”. Neutravidin-agarose (ThermoFisher) was then applied to capture proteins that integrated the labeled fucose by IP. Samples were incubated ON at 4 °C with end-over-end rotation. The next day, neutravidin-captured proteins were washed in 0.1% Triton X-100 and run for analysis by Western blot.

## 3. Results

### 3.1. UEAI Recognizes Intracellular Fucosylated Proteins

**Figure 1:**
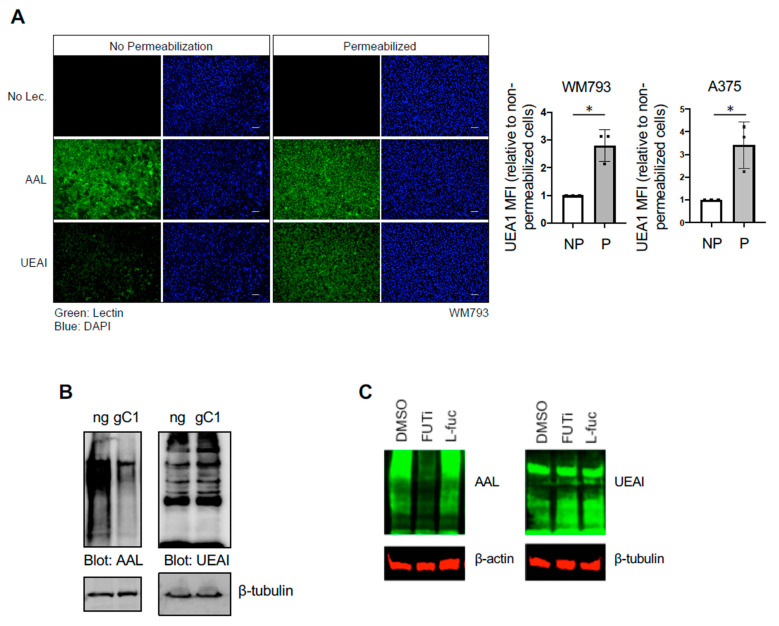
UEAI recognizes intracellular proteins. (**A**) WM793 cells were fixed and permeabilized with Triton X-100 or left unpermeabilized. Cells were subsequently stained with DAPI nuclear stain, FITC-AAL, FITC-UEAI, or no lectin as a control. Cell were visualized by IF for qualitative lectin staining (left panel). For quantification, WM793 and A375 cells were stained as above and analyzed by flow cytometry (right panel; MFI: Mean Fluorescence Intensity (of UEA1 per cell)). * *p* = 0.006 (**B**) Cells transduced and selected with lentivirus containing Cas9/no guide (ng) or Cas9/sgSLC35C1 (gC1) were grown in normal growth media and harvested for protein lysate. Lysates were probed with AAL and UEAI lectin to determine global levels of Golgi-dependent fucosylation. (**C**) WM793 cells were cultured for three days in 2-fluorofucose (2-FF, FUTi, 250 µM), L-fucose (L-fuc, 250 µM), or control. Cells were harvested for protein lysate and blotted for global AAL and UEAI levels.

Studies by our group and others have shown that fucosylation recognized by UEAI lectin is decreased in advanced melanoma relative to primary disease [6], suggesting that alterations in fucosylation recognized by UEAI contributes to disease development or progression and/or to being selected against in a cell autonomous or non-autonomous manner (e.g., tumor immune editing). However, which specific proteins are recognized by UEAI and how fucosylation influences their function and downstream cellular processes or signaling pathways are not known. We characterized the proteins that are recognized by UEAI through lectin pulldown (PD) and subsequent analysis by mass spectrometry (Table S1). Cells were lysed, and proteins that were recognized by UEAI were isolated by incubating lysate with agarose conjugated UEAI lectin prior to analysis. Interestingly, the proteins identified were predominantly intracellular proteins. We confirmed that UEAI recognizes intracellular proteins in WM793 or A375 human primary melanoma cell lines by staining with fluorescently labeled fucose-binding lectins under conditions where the cells were or were not permeabilized (Figure 1A, left). UEAI recognized almost no cell surface proteins and/or glycosylated lipids, as indicated by low or no fluorescence in nonpermeabilized cells, whereas AAL, a lectin with binding affinity for structurally different fucosylated glycans (Consortium for Functional Glycomics), readily recognized fucosylated cell surface substrates. Following permeabilization, UEAI staining was increased, whereas AAL staining was largely unchanged. Quantification by flow cytometry using FITC-conjugated UEAI confirmed that UEAI staining was increased following permeabilization in WM793 as well as A375 melanoma cell lines (Figure 1A, right). These data suggest that UEAI specifically recognizes intracellular fucosylated glycans in melanoma cells.

Fucosylation generally occurs on cell surface and secreted proteins and is dependent on entry into the ER–Golgi network, where fucosyltransferases are present [9,10]. We found that UEAI-recognized fucosylation is not dependent on the ER–Golgi network or the de novo fucose synthesis pathway (Figure 1B,C, respectively). To test whether UEAI fucosylation is dependent of the ER–Golgi network, we knocked out the Golgi GDP-fucose transporter SLC35C1 and tested global fucosylation as recognized by AAL and UEAI. We observed a global decrease in AAL-recognized fucosylation, as indicated by a decrease in AAL signal across glycosylated proteins of various molecular weights. In contrast, UEAI levels did not decrease, indicating the proteins recognized by this lectin are not dependent on fucosylation occurring in the ER–Golgi network or do not enter the secretory pathway. Inhibiting cellular fucosylation using the fucose analog 2-fluorofucose (2-FF) similarly did not have any effect on UEAI-recognized fucosylation, whereas AAL recognized fucosylation was substantially diminished (Figure 1C). These findings suggest that fucosylation of intracellular proteins can occur and that this species of fucosylation is or can be independent of the canonical fucosylation pathway.

### 3.2. RPS3 Is Fucosylated in Normal Tissue and Cancer Cells

**Figure 2:**
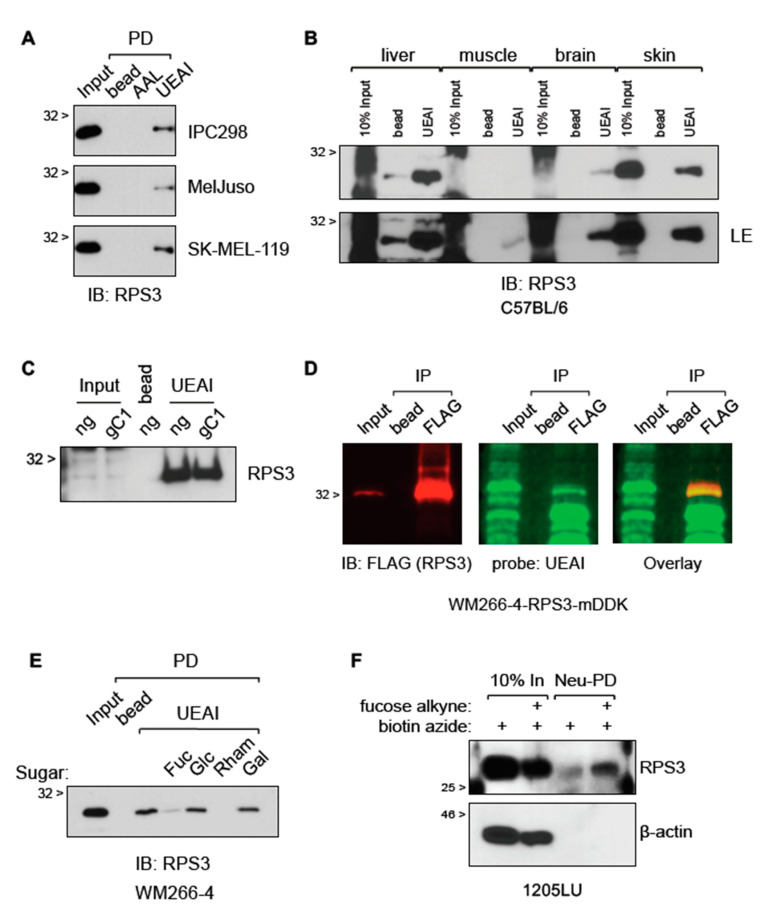
RPS3 is fucosylated in vitro and in vivo. (**A**) RPS3 is recognized by UEAI lectin by agarose-bound lectin pull-down (PD). Cell lines IPC298, MelJuso, and SK-MEL-119 were cultured in growth media and harvested for protein lysate. Lysates were incubated with bead (agarose bead control), AAL (AAL-agarose), or UEAI (UEAI-agarose) to bind fucosylated proteins. UEAI-recognized RPS3 in all three cell lines. (**B**) Liver, muscle, brain, and skin samples were harvested from a C57BL/6 mouse and lysed using a Dounce homogenizer. Lysates were subjected to UEAI PD to isolate UEAI-recognized proteins. RPS3 was recognized by UEAI in all tissue types. LE: long exposure. (**C**) Cells transduced and selected with lentivirus containing Cas9/no guide (ng) or Cas9/sgSLC35C1 (gC1) were grown in normal growth media and harvested for protein lysate (Figure 1B). Lysate were subjected to UEAI PD to isolate UEAI-recognized proteins. Knockout of the Golgi fucose transporter SLC35C1 did not affect the ability of UEAI to recognize RPS3. (**D**) Cells expressing myc-DDK (FLAG)-tagged RPS3 (RPS3-mDDK) were harvested for FLAG IP and Western blot. The membrane was probed with mouse anti-FLAG/anti-mouse-680 and UEAI-biotin/strep-800 to assess direct recognition of RPS3 by UEAI (signal overlap by LI-COR imaging). UEAI recognizes RPS3. (**E**) Cells were grown in normal growth media and harvested for UEAI PD. The lectin PD was supplemented with either no sugar (control), L-fucose (Fuc), glucose (Glc), rhamnose (Rham), or galactose (Gal) at 1 M final concentration. Analysis by Western blot showed that fucose and rhamnose interfere with UEAI lectin recognition of RPS3, whereas glucose and galactose did not affect lectin binding. (**F**) Cells grown in the presence of labeled fucose were analyzed to assess labeling of RPS3. Labeled lysates or unlabeled lysates (control) were subjected to neutravidin pull-down (Neu-PD) to isolate proteins that integrated the labeled fucose. RPS3 did integrate the labeled fucose. β-actin, a non-fucosylated protein, did not show any integration of the labeled fucose (negative control).

Gene Ontology analysis of the UEAI-recognized proteins suggested that ribosomal proteins are overrepresented and thus were pursued for follow-up analysis (Figure S1). Ribosomal protein S3 (RPS3) was selected for further validation and analysis as it had the highest number of unique spectra among ribosomal proteins identified. RPS3 has been reported to have a role in progression in several cancer types [11,12,13,14,15]. We first confirmed and characterized RPS3 expression in tissues and cells. Immunofluorescent staining analysis of a melanoma tumor microarray (TMA) revealed that total RPS3 protein levels increase with stage in melanoma patient samples (Figure S2A). RPS3 is increased in melanoma cell lines relative to primary human epidermal melanocytes and is also increased in drug-resistant isogenic cell lines relative to drug-sensitive parental lines (Figure S2B,C). UEAI PD followed by RPS3 immunoblot (IB) suggested that RPS3 is fucosylated in a number of melanoma cell lines as well as in normal mouse tissue homogenates (Figure 2A,B). We observed that the amount of fucosylated RPS3 appears to generally correlate with total level of RPS3, independent of tissue type—tissues that showed higher relative levels of RPS3 (e.g., skin) also had higher amounts of fucosylated RPS3 relative to tissues with lower total expression (e.g., muscle required long exposure (LE) time to see the fucosylated band). Knockout of SLC35C1 did not affect the recognition of RPS3 by UEAI as assessed by UEAI PD (Figure 2C), in agreement with our finding that SLC35C1 knockout did not affect global levels of fucosylation recognized by UEAI (Figure 1B). We next tested whether UEAI directly recognizes RPS3 to rule out the possibility that RPS3 is indirectly pulled down by UEAI through indirect interactions. We immunoprecipitated (IP) RPS3 and subsequently co-probed a Western blot for RPS3 and UEAI to assess if there was direct overlap of RPS3 and UEAI signal by LI-COR analysis (Figure 2D). We observed direct overlap of RPS3 and UEAI, confirming that UEAI directly recognized RPS3. UEAI PD of RPS3 was abrogated in the presence of excess exogenous L-fucose or rhamnose (a structurally similar 6-deoxy-sugar), whereas incubation with glucose or galactose did not affect lectin recognition (Figure 2E). We further confirmed direct fucosylation of RPS3 by culturing cells with L-fucose alkyne, allowing integration of this labeled form of L-fucose into cellular glycans for 2 days and then lysing the cells and isolating fucosylated proteins using a click chemistry approach [16] to covalently link the L-fucose alkyne species to biotin azide for downstream isolation using neutravidin beads. This approach has the advantage of being independent of UEAI lectin and uses an approach to directly label fucosylated proteins using a synthetic chemistry strategy. Analysis by Western blot confirmed RPS3 is indeed directly fucosylated (Figure 2F).

### 3.3. Fucosylated RPS3 Is Associated with Proteins That Control RNA Splicing, Trafficking, and Stability

**Figure 3:**
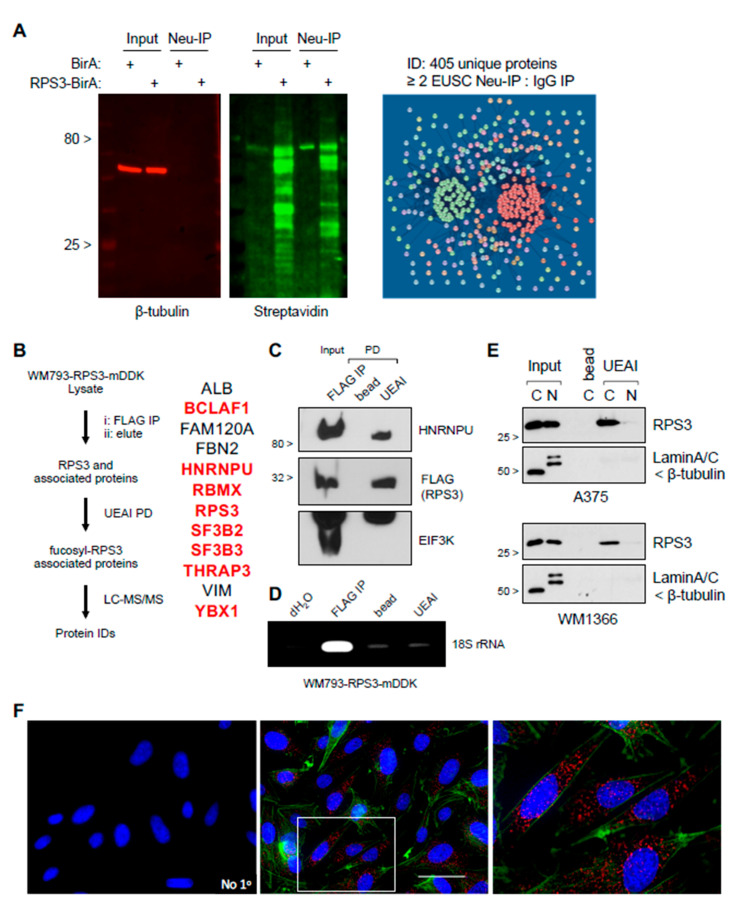
Fucosylated RPS3 interacts with a ribosome-independent group of proteins associated with posttranscriptional control of RNA. (**A**) RPS3 BioID lysates analyzed by Western blot to show specific biotin labeling of proteins and Neu-IP capture in cells expressing RPS3-BirA versus BirA expression alone. Neu-IP captured proteins identified by LC-MS/MS analysis were found to segregate into three groups by STRING analysis: (i) ribosome-associated proteins (red), (ii) posttranscriptional RNA regulators (green), and (iii) proteins not strongly associated with either group (i) or (ii). (**B**) Workflow to identify proteins associated specifically with fucosylated RPS3. LC-MS/MS identified 12 proteins, a majority of which are known to interact amongst each other (highlighted in red) and posttranscriptionally regulate RNA. (**C**) Validation of interaction between RPS3 and HNRNPU. Analysis of FLAG IP elution of RPS3-mDDK (step 2 in panel B) and subsequent UEAI-PD for HNRNPU, FLAG (RPS3), and EIF3K (to confirm ribosome-independent interaction). (**D**) RNA immunoprecipitation analysis of FLAG IP elution and subsequent UEAI-PD was assessed for interaction with small ribosomal RNA (18S rRNA). (**E**) Cells were harvested for cytoplasmic (C) and nuclear (N) fractions prior to UEAI-PD to determine localization of fucosylated RPS3. Lamin A/C and β-tubulin were probed as markers for the nuclear and cytoplasmic fraction, respectively. (**F**) Cells were grown on glass coverslips and fixed for proximity ligation analysis (PLA) of RPS3 and UEAI lectin (Red). Cells were stained with phalloidin (Green) to visualize the cytoplasmic region and DAPI to visualize the nuclear region. PLA with no primary antibodies (No 1°) was run as a negative control. RPS3-UEAI-positive foci were found to be localized primarily to the cytoplasmic fraction.

Although RPS3 is primarily known for its role in ribosome assembly and protein translation [17], the protein is also known to have ribosome-independent functions that are controlled through other posttranslational modification [18,19,20,21,22,23]. We therefore hypothesized that glycosylation/fucosylation might mark RPS3 for a ribosome-independent function. Using an RPS3-biotinylation identification (BioID) approach to label proteins that are stably or transiently associated with RPS3 for subsequent isolation and identification (see Materials and Methods) [24], we found that RPS3 segregates into two primary putative functional species: (i) a species that strongly associates with ribosomal proteins, representing ribosomal RPS3; and (ii) a species that strongly segregates with non-ribosomal proteins that are associated with RNA splicing, trafficking, and stability (Figure 3A, Figure S3 and Table S2). To characterize which functional cluster fucosylated RPS3 associates with, we conducted experiments to immunoprecipitate ectopically expressed myc-DDK-tagged RPS3 from cell lysates, elute RPS3, and its binding partners using FLAG peptide and subsequently to isolate fucosylated RPS3 and associated proteins by UEAI PD (Figure 3B). Mass spectrometric analysis identified 12 proteins, including RPS3. Fucosylated RPS3 was found to interact with proteins associated with posttranscriptional regulation of RNA but not with any other ribosomal proteins. We were also unable to detect coimmunoprecipitation of EIF3K or 18S rRNA with fucosylated RPS3, strongly suggesting that fucosylated RPS3 is independent of the ribosome (Figure 3C,D). Several reports have characterized nuclear functions for RPS3 that are controlled through posttranslational phosphorylation (e.g., nuclease activity in DNA repair [19,20]). We therefore tested whether fucosylated RPS3 is localized to the nucleus or cytoplasm. Fucosylated RPS3 was found only to localize to the cytoplasmic fraction, as assessed by either biochemical subcellular fractionation followed by UEAI PD or by lectin-mediated proximity ligation assay (PLA), a modified PLA protocol developed in our laboratory to specifically characterize levels and subcellular localization of the fucosylated species of specific proteins (Figure 3E,F). Interaction with heterogenous ribonucleoprotein U (HNRNPU) was selected for further analysis as this protein is reported to play roles in regulating RNA stability in both the nucleus and cytoplasm [25]. We found that HNRNPU does localize to the cytoplasmic fraction by both subcellular fractionation and immunofluorescence analysis, and its steady state protein levels are also increased in melanoma cell lines relative to primary cells (Figure S4A,B), consistent with those of RPS3. We subsequently validated interaction of fucosylated RPS3 with HNRNPU by co-IP analysis (Figure S4C,D)—cells expressing myc-DDK-tagged RPS3 were subjected to FLAG IP, RPS3 elution, followed by UEAI PD to isolate fucosylated RPS3 (as described above). Analysis by Western blot showed that HNRNPU was co-immunoprecipitated with fucosylated RPS3. Interestingly, we found that the interaction of HNRNPU and fucosylated RPS3 is dependent on RNA: coimmunoprecipitation of HNRNPU and RPS3 was abolished when pre-incubating cell lysates with RNaseA, suggesting that fucosylated RPS3 and HNRNPU posttranscriptionally co-regulate a subset of cellular RNAs (Figure S4E).

**Figure 4:**
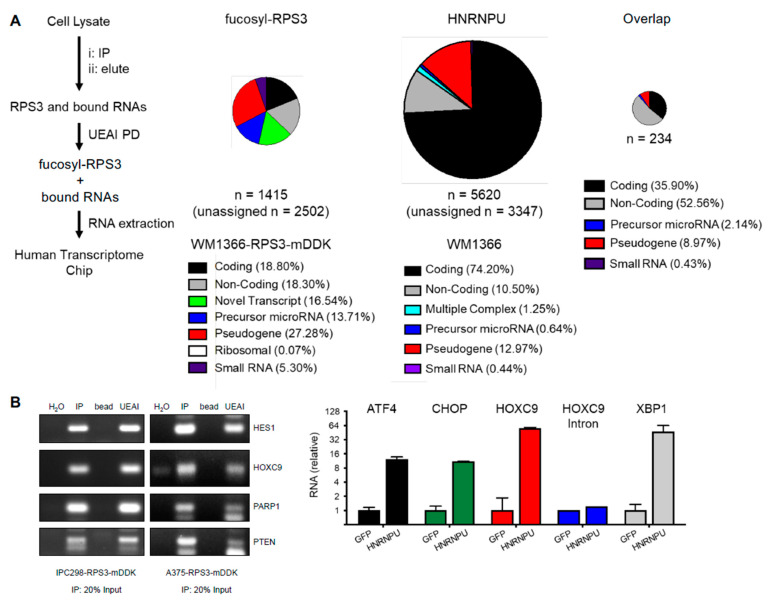
Identification of RNAs that interact with fucosylated RPS3 and HNRNPU. (**A**) Workflow and results for fucosylated RPS3 (fucosyl-RPS3) and HNRNPU RIP-Chip. RNAs isolated by fucosylated RPS3 PD, HNRNPU IP, or IgG control IP were analyzed on a transcriptome-level expression array chip. RNAs detected at > 2-fold higher in fucosylated RPS3 PD or HNRNPU IP relative to IgG control IP were counted as a specific interaction. Results were analyzed to assign RNAs to one of several distinct subclasses of RNA. (**B**) The RNAs identified in the RIP-Chip were validated by RIP-PCR and RIP-qPCR. Coding RNAs for HES1, HOXC9, PARP1, and PTEN were validated by PCR for the fucosylated RPS3 RIP (left). Coding RNAs for ATF4, CHOP, HOXC9, and XBP1 were validated by qPCR for interaction with HNRNPU. HOXC9 intron was tested to determine whether HNRNPU interacts with mature RNAs, as opposed to unprocessed, pre-spliced transcripts.

To identify the RNAs that interact with fucosylated RPS3 and HNRNPU, we isolated RNAs from RNA-IP (RIP) experiments for fucosylated RPS3 and HNRNPU and subjected those RNAs to analysis by Chip (RIP-Chip) (Figure 4A). Fucosylated RPS3 was found to interact with nearly 4000 cellular RNAs, approximately 80% of which are for non-coding RNA species. The findings (i) that mRNAs accounted for only approximately 20% of the RNAs bound by fucosylated RPS3 and (ii) that ribosomal RNAs are nearly absent from the dataset support a ribosome-independent function for fucosylated RPS3 in the posttranscriptional regulation of RNAs. HNRNPU bound nearly 9000 RNAs, approximately 75% of which are coding, consistent with previous reports that HNRNPU binds to pre-mRNAs and mature mRNAs in the nucleus and cytoplasm, respectively [26]. Fucosylated RPS3 and HNRNPU co-bound 234 RNAs, a majority of which are non-coding RNAs. Several mRNAs identified in each dataset were validated by RIP-PCR (fucosylated RPS3) or RIP-qPCR (HNRNPU) (Figure 4B).

### 3.4. RPS3 Fucosylation Responds to MAPK Inhibition in Melanoma Cells

**Figure 5:**
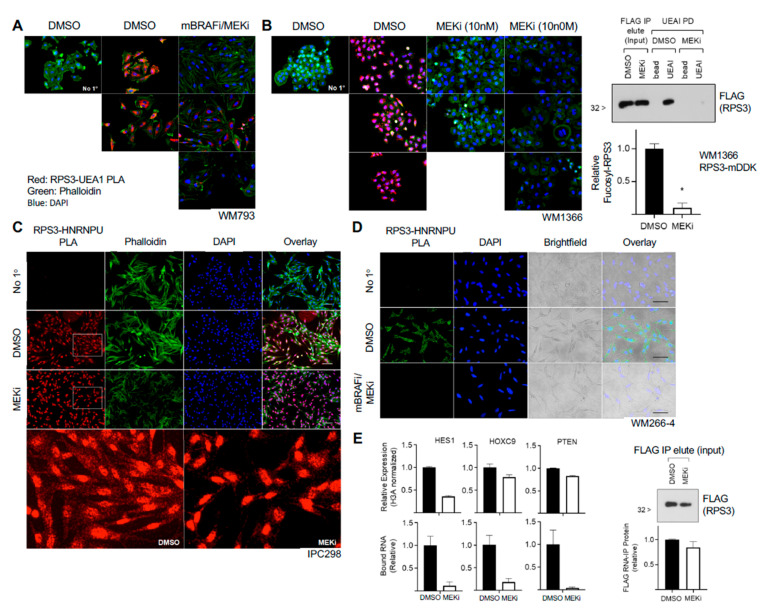
MAP kinase inhibitors decrease RPS3 fucosylation and interaction with RNA. (**A**) Treatment of BRAF-mutant cells with combination mutant-BRAF inhibitor (mBRAFi, PLX4032, 1 µM) and MEK inhibitor (MEKi, trametinib, 10 nM) decreased RPS3 fucosylation as assessed by PLA (red). Cells were grown on glass coverslips and treated for 2 d with inhibitors prior to fixation and analysis. Cells were counterstained with phalloidin (green) and DAPI (blue). No primary (No 1°) was run as a negative control. Several representative fields are shown. (**B**) NRAS-mutant cells were grown on glass coverslips and treated with MEKi (10 nM, 100 nM) for 2 d prior to fixation and analysis for fucosylated RPS3 (red). Cells were counterstained with phalloidin (green) and DAPI (blue). No primary (No 1°) was run as a negative control. Several representative fields are shown. RPS3 IP, elution, and subsequent UEAI-PD on MEKi-treated lysates was conducted to confirm a decrease in RPS3 fucosylation by Western blot. Cells were treated with 10 nM MEKi for 2 d prior to lysis for downstream analysis. Results were analyzed by *t*-test (*n* = 2). (**C**) NRAS-mutant cells were grown on glass coverslips and treated with MEKi (10 nM) or control for 2 d prior to analysis for interaction between RPS3 and HNRNPU by PLA (red). Cells were counterstained with phalloidin (green) and DAPI (blue). No primary (No 1°) was run as a negative control. (**D**) BRAF-mutant cells were grown on glass coverslips and treated with combination mBRAFi (1 µM) and MEKi (10 nM) for 2 d prior to analysis for interaction between RPS3 and HNRNPU as assessed by PLA (green). Cells were counterstained with DAPI (blue) and also visualized by brightfield to observe cell morphology. No primary (No 1°) was run as a negative control. (**E**) NRAS-mutant cells were treated with MEKi (10 nM) or control for 2 d prior to analysis for relative mRNA level and relative binding to fucosylated RPS3. *HES1*, *HOXC9*, and *PTEN* were analyzed. Relative expression was normalized to *H3A.* A fraction of the UEAI-PD input (following RPS3 IP and elution) was taken and analyzed by Western blot as a control for IP efficiency between control and MEKi-treated groups (lower panel). * *p* < 0.05.

**Figure 6:**
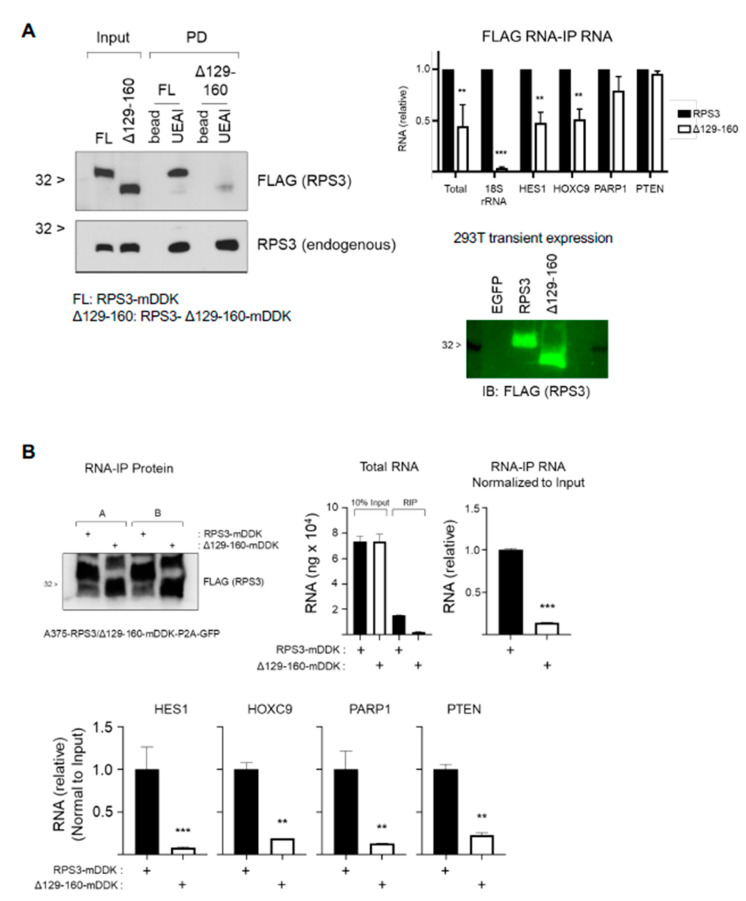
RPS3 fucosylation mutant shows impaired ability to interact with RNA. (**A**) Fucosylation mutant RPS3 missing amino acids 129—160 (RPS3-Δ129-160-mDDK) was transfected into 293T for 2 d prior to harvest for protein lysate. Lysates were subsequently analyzed for RPS3 fucosylation by UEAI-PD and Western blot. Membranes were probed for FLAG to detect the deletion mutant and for RPS3 to detect endogenous RPS3. The ability of the mutant to interact with RNA was tested by FLAG RIP and subsequent RNA purification and analysis by qPCR. A sample from FLAG RIP was taken for protein analysis to confirm equal amount of RPS3 was immunoprecipitated. Results were analyzed by 2-way ANOVA (*n* = 2). (**B**) A375 cells expressing either RPS3-mDDK- or RPS3-Δ129-160-mDDK-P2A-GFP were sorted on GFP+ cells, grown back up and immediately harvested for FLAG RIP analysis. A sample from FLAG RIP was taken for protein analysis to confirm equal amount of RPS3 was immunoprecipitated. Results were analyzed by 2-way ANOVA (*n* = 2). ** *p* < 0.01; *** *p* < 0.001.

To gain insight into how fucosylation of RPS3 may impact protein function, we tested by lectin-mediated PLA and Western blot whether fucosylation level responds to stimulus in melanoma cells. Here we used the mitogen-activated protein kinase (MAPK) pathway inhibitors vemurafenib (PLX4032) (mutant BRAF inhibitor, mBRAFi) and trametinib (MEK inhibitor, MEKi) as these are frontline therapeutics in the management of melanoma in the clinic. Treatment of mBRAF melanoma cells with combination mBRAFi/MEKi led to a decrease in fucosylated RPS3 as assessed by PLA and UEAI lectin PD (Figure 5A). A similar finding was observed in mutant NRAS cells treated with MEKi (Figure 5B). The decrease in RPS3 fucosylation was neither attributable to a global decrease in fucosylation recognized by UEAI lectin nor to a decrease in total RPS3 protein levels (Figure S5A,B). MAPKi did not decrease UEAI levels globally as assessed by Western blot, whereas AAL level was increased in response to these inhibitors. The decrease in fucosylated RPS3 was also not associated with reduced RPS3 protein levels. Decreased fucosylation of RPS3 in response to treatment was associated with a decrease in binding to several RNAs we identified by RIP-Chip and a decrease in interaction with HNRNPU (Figure 5C–E). Fucosylated RPS3 showed decreased binding to HES1, HOXC9, and PTEN mRNAs following treatment with MEKi relative to total mRNA level. Interaction of total RPS3 with HNRNPU showed a decrease in the cytoplasmic fraction of NRAS mutant melanoma cells treated with MEKi and in BRAF mutant cells treated with combination mBRAFi/MEKi as assessed by PLA analysis. The decrease in interaction could not be attributed to changes in protein level or localization (Figure S5C). These data suggest that fucosylation of RPS3 influences its ability to interact with RNAs and HNRNPU. To test this hypothesis, we attempted to identify the site(s) that are fucosylated on RPS3 through a mutagenesis and UEAI lectin PD approach and then tested whether the fucosylation-defective mutant showed an impaired ability to interact with RNAs and HNRNPU. We generated 14 serine or threonine RPS3 point mutants, 1 compound mutant, and 8 deletion mutants (not shown) for this analysis and found only 1 deletion mutant that abolished UEAI binding: ∆129-160 (Figure 6A, left). RIP analysis of the deletion mutant showed a defect in ability to interact with RNAs (Figure 6A, right), suggesting that fucosylation of RPS3 plays a role in mediating its interaction RNAs (Figure 6B). Although this deletion abolished RPS3 fucosylation, the region was also found to be required for stability (Figure S6A). The ∆129-160 RPS3 mutant, which lacks amino acids 129—160 (a region containing 4 serine residues potentially subject to glycosylation), is rapidly degraded through the proteasome pathway, as protein level could be rescued by incubating cells with MG132 (a proteasome inhibitor) (Figure S6A), and no stable mutant-expressing cell line could be produced for phenotypic testing.

## 4. Discussion

Glycosylation is known to play an important role in influencing protein–protein interactions, particularly in how cells interact with proteins in the extracellular environment. Glycosylation of extracellular receptors, secreted proteins, or exogenous ligands can influence downstream cellular signaling pathways and in turn impact cellular behavior (e.g., adhesion/migration). Due to the spatial restriction of fucosyltransferase enzymes to the secretory network, fucosylation has generally been thought to only influence proteins and signaling pathways, and thus cellular behavior, through modification of extracellular receptors and/or secreted proteins. Aside from posttranslational N-acetylglucosylation (GlcNAC), a reversible posttranslational modification on intracellular proteins with a GlcNAC moiety, there is no clearly recognized role for other intracellular glycosylation events that can influence protein function and/or cellular signaling pathways and downstream cellular behavior. In this report, we find that a large number of intracellular proteins are potentially posttranslationally modified with L-fucose. An unbiased UEAI lectin screen for fucosylated proteins identified only intracellular proteins, and we subsequently validated RPS3 as fucosylated using an approach for labelling fucosylated proteins with a fucose analog that allows for click chemistry-mediated identification of proteins that are posttranslationally fucosylated.

We find that fucosylation of RPS3 appears to be independent of the canonical fucosylation pathway. Knockout of the Golgi GDP-L-fucose transporter SLC35C1 had no effect on fucosylation of RPS3 or global UEAI-recognized fucosylation, and inhibition of global GDP-L-fucose synthesis with 2-FF also did not affect UEAI-recognized fucosylation. These data suggest that fucosylation outside of the ER–Golgi network and de novo GDP-L-fucose synthesis pathway can and does occur and that there are uncharacterized pathway(s) that control fucosylation on intracellular proteins that may be independent of the canonical fucosylation pathway. It is possible that SLC35C1 knockout or 2-FF are insufficient to entirely block fucosylation (i.e., compensation by SLC35C2), and retrograde trafficking/transport represents one mechanism that could result in cytoplasmic localization of fucosylated RPS3. However, as a majority of our in vitro studies have been performed in melanoma cell lines, it is also possible that the aberrant expression of splice variants of fucosyltransferases that are no longer restricted in localization to within the ER–Golgi network mediate RPS3 fucosylation. Further studies are required to test this possibility. 

Fucosylation of RPS3 is associated with a species of RPS3 that is independent of the ribosome, and we also found that fucosylation level can respond to stimulus (MAPKi). This suggests that fucosylation of RPS3 is regulated and influences protein function, either tagging a pool of ribosome-independent RPS3 for interaction with a protein complex that posttranscriptionally regulates RNAs or excluding a pool of RPS3 from the ribosome and/or nucleus. The stress-/therapeutic agent-responsive nature of this mechanism in melanoma suggests potential pathogenic contributions that will require further study. We were unable to identify an individual specific site where RPS3 is fucosylated (or glycosylated), and a deletion mutant that abolished fucosylation on RPS3 could not be stably expressed, as it compromised protein stability. The finding that several deletion mutants of RPS3 can be expressed and are glycosylated, whereas the single deletion mutant identified that cannot be fucosylated is rapidly degraded, suggests that this region is both important for protein stability and glycosylation. These findings support the notion that the fucosylation or glycosylation of RPS3 is not necessarily site-specific but is instead spread across multiple sites within a region. This might be indicative of association with a specific protein complex and glycosylation by close proximity to glycosyltransferase enzymes. Further work is needed to clarify the mechanism by which RPS3 is fucosylated, how it influences its function, and how fucosylation can occur outside of the canonical fucosylation pathway. Subsequent elucidation of such non-canonical fucosylation events is anticipated to provide important insight into how fucosylation, and defects therein, might significantly contribute to cancer pathology and therapeutic responsiveness.

## Data Availability

The data presented in this study are available in Tables S1 and S2.

## Supplementary Materials

The following are available online at https://www.mdpi.com/article/10.3390/cells10061310/s1, Figure S1: Gene Ontology analysis highlights proteins involved in translation as over-represented in UEAI-recognized fucosylated proteins, Figure S2: High RPS3 is associated with advanced disease and drug resistance in melanoma samples, Figure S3: Enlarged STRING analysis of RPS3-interacting proteins, Figure S4: Fucosylated RPS3 interacts with HNRNPU in an RNA-dependent manner, Figure S5: MAP kinase inhibitor treatment does not affect global UEAI levels or RPS3 or HNRNPU localization, Figure S6: RPS3 fucosylation mutant shows decreased stability and is rapidly degraded, Table S1: UEAI-recognized proteins in melanoma cells, Table S2: RPS3-interacting proteins identified by BioID.
