# Supplementary material for: Fucosylated Proteome Profiling Identifies a Fucosylated, Non-Ribosomal, Stress-Responsive Species of Ribosomal Protein S3
Source: Cells. 2021 May 25;10(6):1310. doi: 10.3390/cells10061310 (PMC8228307; doi:10.3390/cells10061310)
Supplement: Supplementary file 1 [file cells-10-01310-s001.zip › supplementary figures.pdf]

Supplementary Figures

Annotation Cluster 1	Enrichment Score 21.21	# of proteins	P_value	Benjamini
GOTERM_BP_DIRECT	translational initiation	30	2.40E-30	2.40E-27
GOTERM_BP_DIRECT	SRP-dependent cotranslational protein targeting to membrane	26	5.70E-29	2.80E-26
GOTERM_BP_DIRECT	nuclear-transcribed mRNA catabolic process, nonsense-mediated decay	26	3.70E-26	1.20E-23
GOTERM_BP_DIRECT	viral transcription	24	6.90E-24	1.70E-21
UP_KEYWORDS	Ribosomal protein	24	2.10E-20	6.00E-19
GOTERM_BP_DIRECT	rRNA processing	26	1.30E-19	2.60E-17
GOTERM_MF_DIRECT	structural constituent of ribosome	25	7.00E-18	4.90E-16
GOTERM_BP_DIRECT	translation	26	7.70E-18	1.30E-15
KEGG_PATHWAY	Ribosome	24	1.10E-17	1.30E-15
GOTERM_CC_DIRECT	ribosome	21	1.30E-16	3.70E-15

Figure S1. Gene Ontology analysis highlights proteins involved in translation as over-represented in UEAI-recognized fucosylated proteins. Proteins with ≥5 exclusive unique spectra count (EUSC) were analyzed using DAVID Bioinformatics Database Functional Annotation tool using default settings.

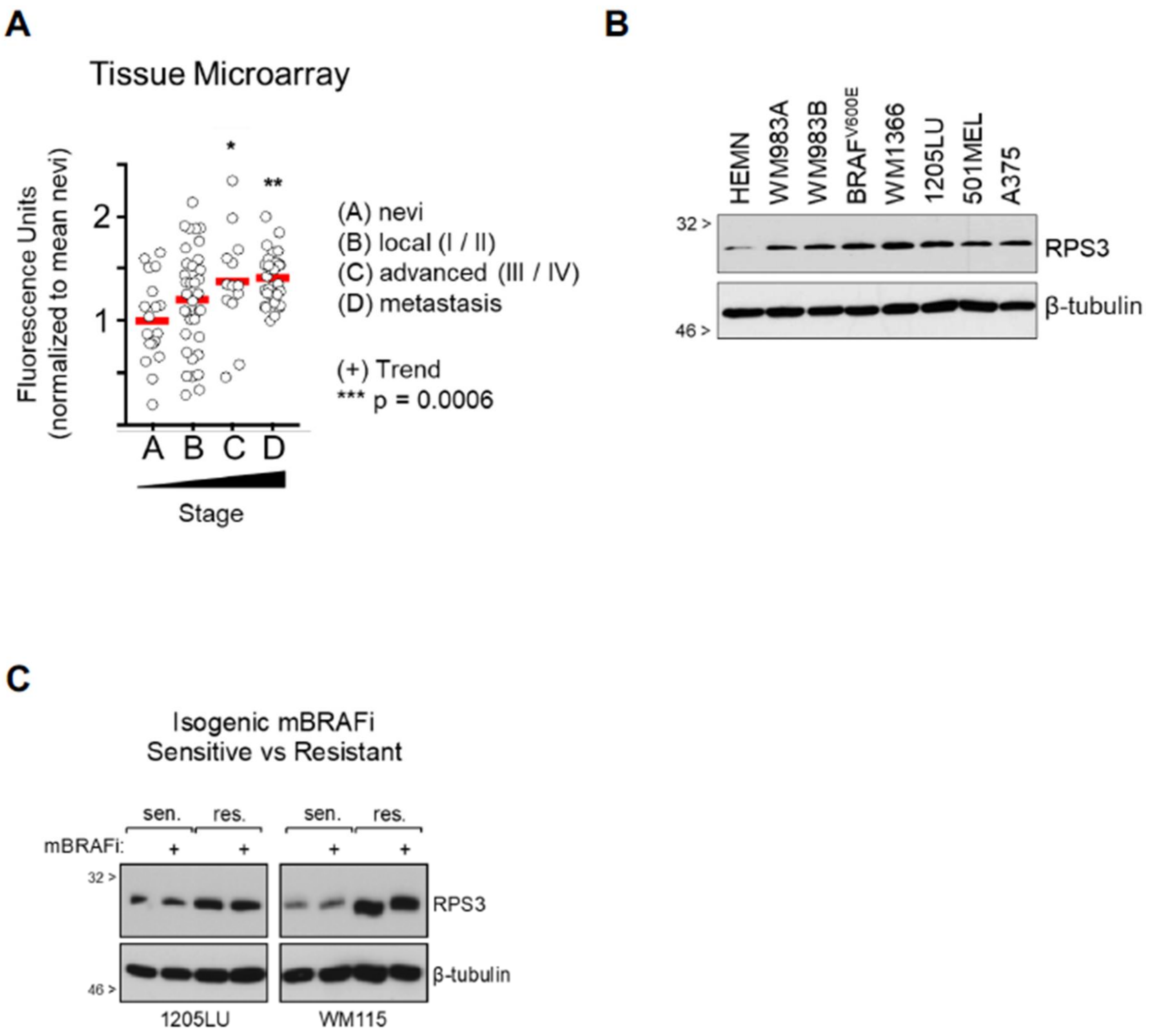

Figure S2. High RPS3 is associated with advanced disease, poorer overall survival, and drug resistance in melanoma samples. (**A**) Melanoma tissue microarray containing nevi, Stage I, II, III, IV, and metastatic samples stained for RPS3. Specimens were divided into four groups: nevi (benign), local (Stage I/II), advanced (Stage III/IV), and metastatic (direct sample of a metastatic growth). Data are normalized to mean nevi signal. Red bar represents the mean for each group. Data was analyzed by 2-way ANOVA. (**B**) The Cancer Genome Atlas (TCGA) melanoma dataset was accessed through cBioPortal.org. RPS3 mRNA level was binned by z-Score: < -1, −1 to 0, 0 to 1, 1 to 2, and >2. The overall survival (OS) was plotted against the corresponding mRNA datapoint to analyze the relationship between survival and RPS3 expression. Red bar represents the mean for each group. Data was analyzed by 2-way ANOVA. (**C**) Primary human epidermal melanocytes (HEMN) and a panel of melanoma cell lines (all other cells shown) analyzed for RPS3 protein level by Western blot. (**D**) Isogenic mutant BRAF inhibitor (mBRAFi, PLX4032, 1 μM) sensitive and resistant cell lines were grown either 3 days off drug or 2 days on drug to assess RPS3 protein level.

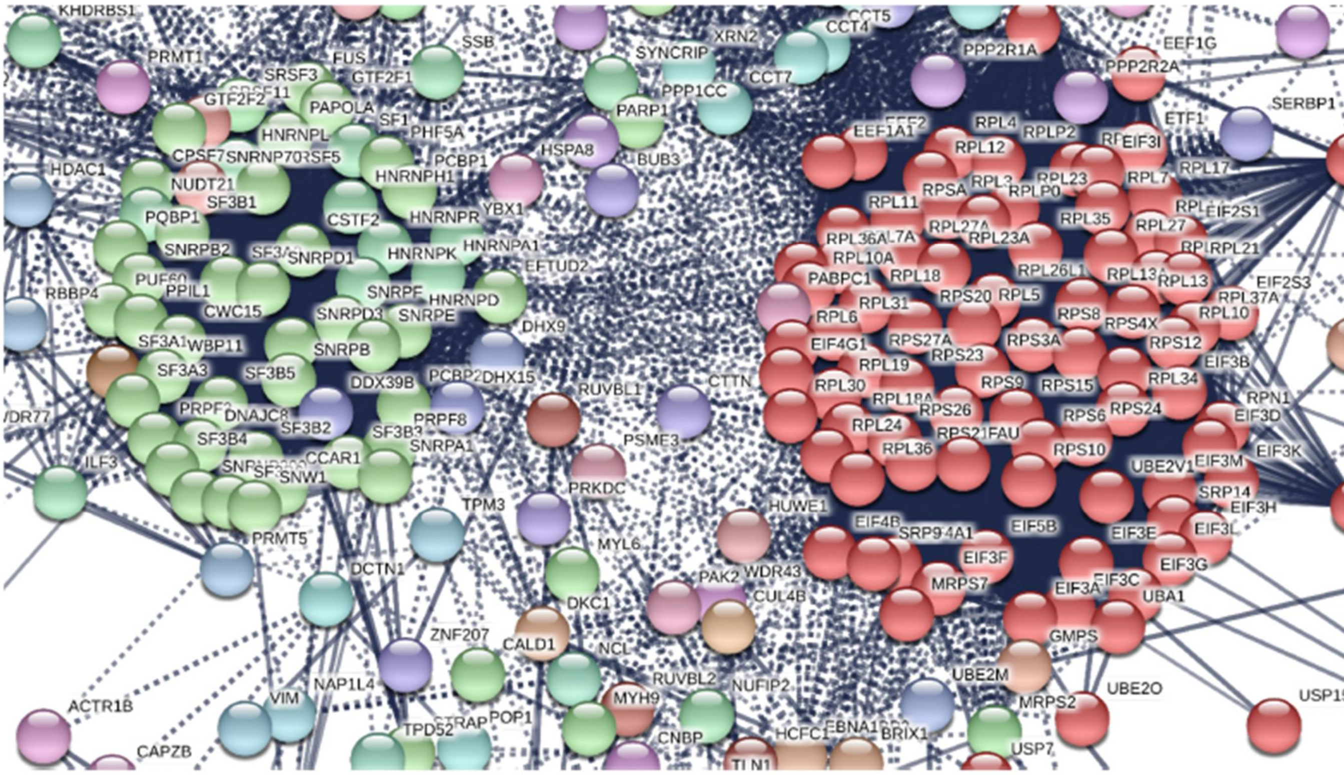

Figure S3. Enlarged STRING analysis of RPS3 interacting proteins. RPS3 BioID analysis identified 405 unique proteins. Analysis by STRING found that proteins segregate into two primary nodes: ribosome-associated proteins (red) and ribosome-independent proteins that post-transcriptionally regulate RNA (green).

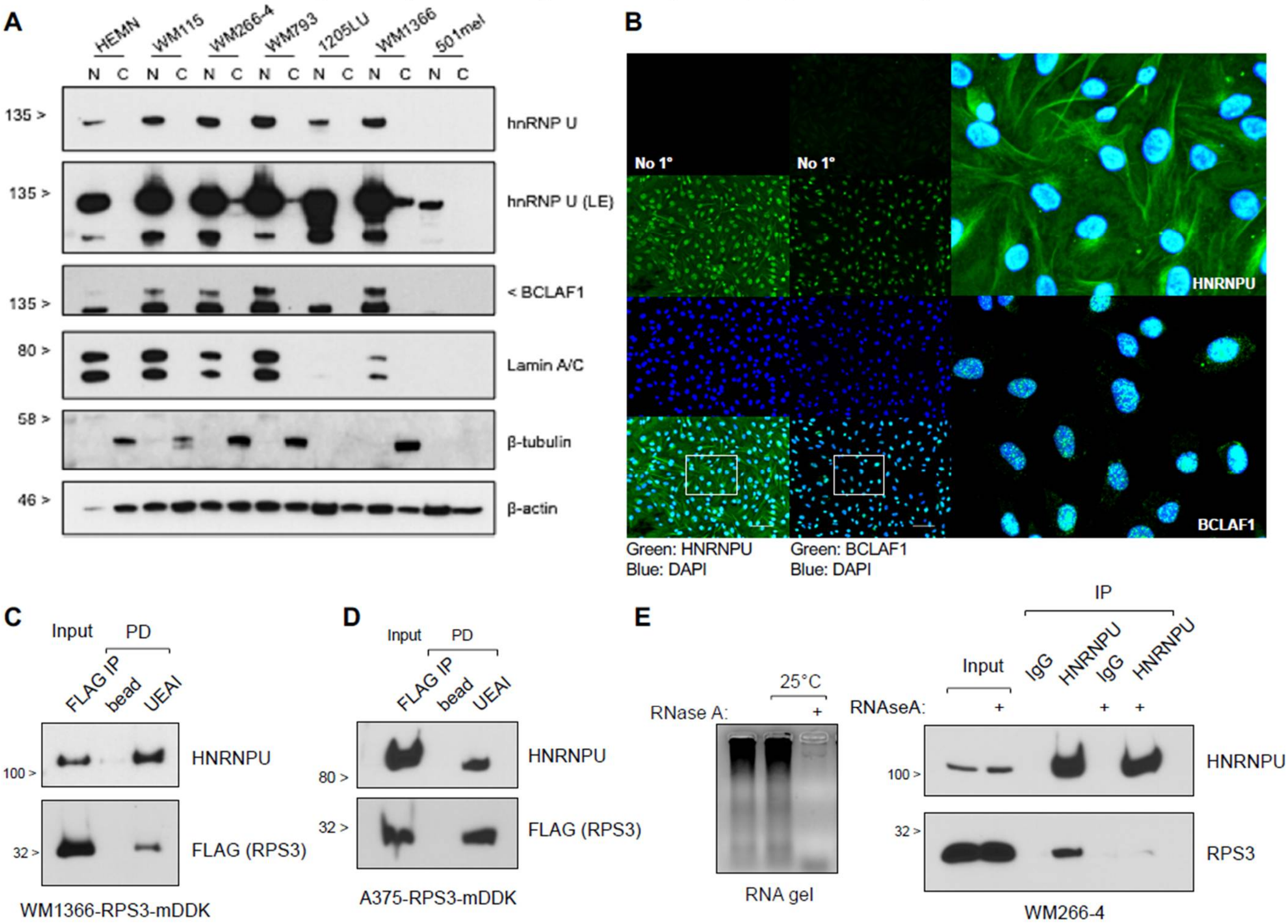

Figure S4. Fucosylated RPS3 interacts with HNRNPU in an RNA-dependent manner. (**A**) Primary human epidermal melanocytes (HEMN) and a panel of melanoma cell lines (all other cells shown) grown and harvested for nuclear (N) and cytoplasmic (**C**) fractions. Lysates were analyzed for protein level and localization of HNRNPU and BCLAF1. (**B**) Cells were grown on glass coverslips and fixed for visualization of HNRNPU and BCLAF1 (green). Cells were counterstained with DAPI to visualize the nuclear region. HNRNPU could be seen in both the extra-nuclear and nuclear space, whereas BCLAF1 was found only in the nuclear region. IF staining with no primary antibody (No 1°) was run as a negative control. (**C**) and (**D**) Cells expressing myc-DDK (FLAG) tagged RPS3 (RPS3-mDDK) were lysed and subsequently subjected to FLAG IP. RPS3 and associated proteins were then eluted and subjected to UEAI-PD to isolate proteins associated with fucosylated RPS3. Fucosylated RPS3 was found to co-IP HNRNPU, confirming interaction. (**E**) RPS3 and HNRNPU interaction was tested for dependence on the presence of RNA. Cell lysates were incubated with RNase A for 1h prior to IP. Lysates were subjected to HNRNPU IP and subsequently analyzed for co-IP of RPS3.

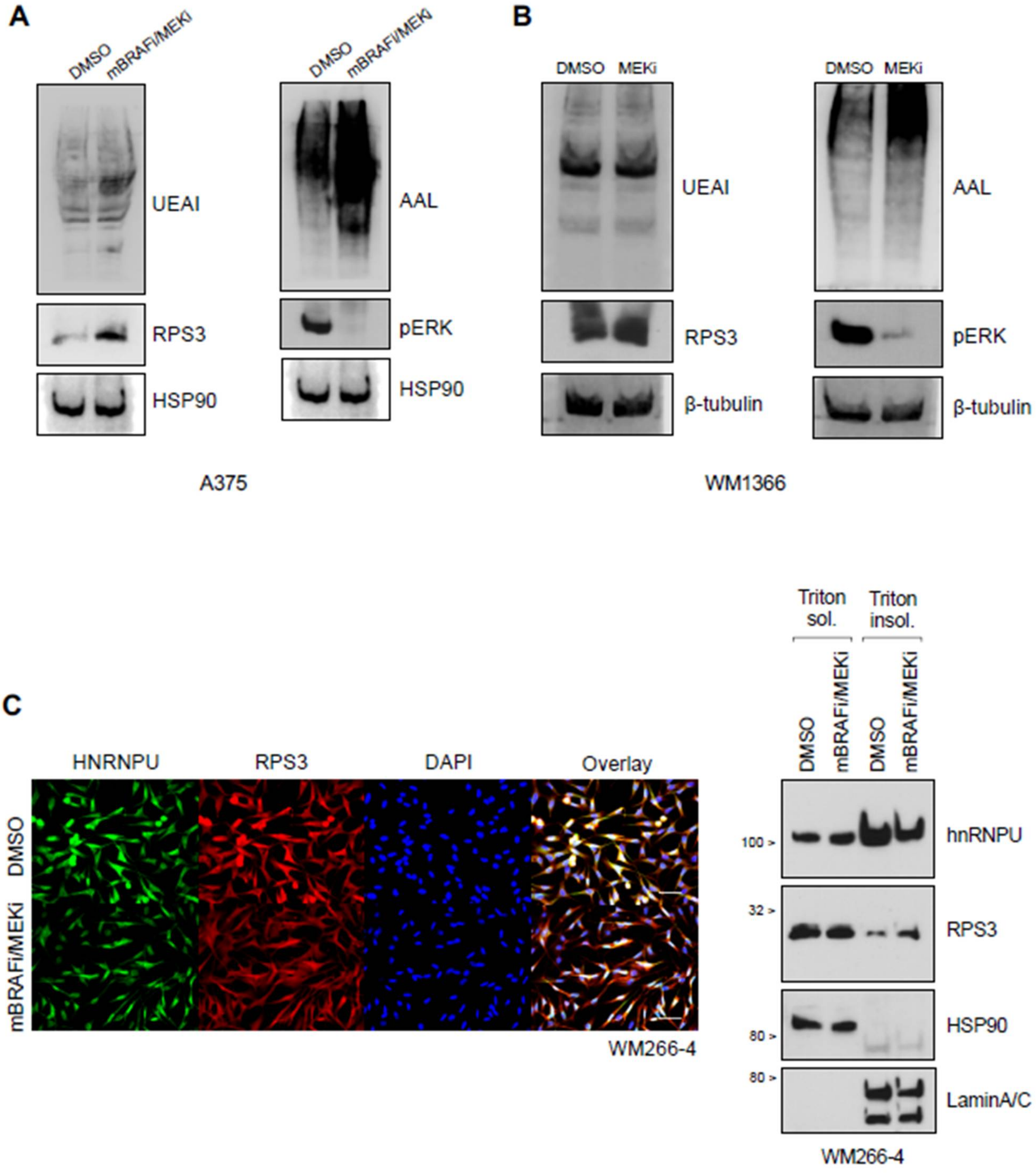

Figure S5. MAP kinase inhibitor treatment does not affect global UEAI levels, or RPS3 or HNRNPU localization. (**A**) Mutant BRAF cells were treated with combination mutant-BRAF inhibitor (mBRAFi, PLX4032, 1 μM) and MEK inhibitor (MEKi, trametinib, 10 nM) for 2 d prior to harvest for analysis of global UEAI protein level. AAL was probed to determine any effect of global golgi-dependent fucosylation level. pERK was probed to confirm MAPK pathway inhibition. (**B**) NRAS-mutant cells were treated with MEKi (10 nM) for 2 d prior harvest for analysis of global UEAI protein level. AAL was probed to determine any effect of global golgi-dependent fucosylation level. pERK was probed to confirm MAPK pathway inhibition. (**C**) BRAF-mutant cells were grown on glass coverslips and treated with combination mBRAFi (1μM) and MEKi (10 nM) for 2 d prior to analysis for RPS3 (red) and HNRNPU (green) localization. Cells were counterstained with DAPI nuclear stain. Cells were similarly treated for analysis of RPS3 and HNRNPU location by fractionation.

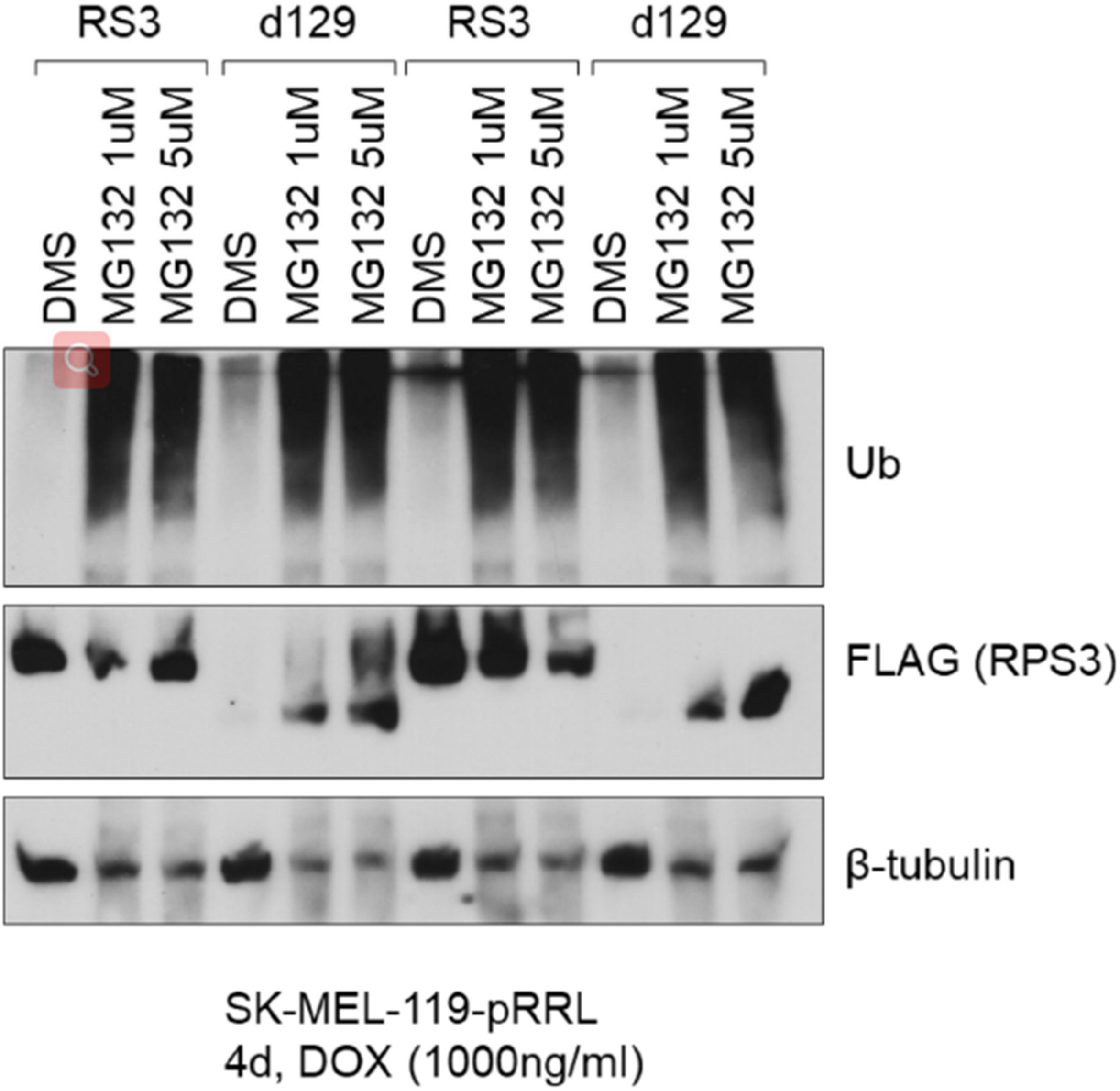

Figure S6. RPS3 fucosylation mutant shows decreased stability and is rapidly degraded. Cells transduced with lentivirus containing DOX inducible EGFP, RPS3-mDDK or RPS3-Δ129-160-mDDK (Δ129). Cells were treated with DOX at 1000 ng/mL for 4 d. On the third day of DOX treatment, cells were put on MG132 at 1 and 5 μM and incubated overnight. Cells were then harvested for analysis by Western blot.
